# Supplementary material for: Effects of Undaria pinnatifida-derived brown algae polysaccharide (UPS) on the nutritional composition, digestive capacity, immune performance and intestinal microbiota of juvenile sea cucumber (Apostichopus japonicus)
Source: PeerJ. 2025 Aug 19;13:e19944. doi: 10.7717/peerj.19944 (PMC12372788; doi:10.7717/peerj.19944)
Supplement: Supplemental Information 1 [file peerj-13-19944-s001.docx]

|  | UPS concentration （groups） | | | | | |
| --- | --- | --- | --- | --- | --- | --- |
|  | HS0 | HS1 | HS2 | HS3 | HS4 | HS5 |
| Initial body weight (W0, g) | 310.03±  3.65 | 317.57±  4.94 | 327.53±  1.99 | 334.9±  13.67 | 334.77±  3.11 | 329.03±  6.84 |
| Final body weight (WT, g) | 359.93±  14.11 | 379.1±  10.78 | 386.57±  2.46 | 384±  24.45 | 382.27±  12.01 | 374.67±  7.62 |
| Weight gain rate (SGR, %) | 16.14±  3.33^a^ | 19.37±  1.21^a^ | 18.02±  0.28^a^ | 14.6±  1.81^a^ | 14.22±  2.64^a^ | 13.93±  2.61^a^ |
| Specific growth rate (SGR, %d) | 0.25±  0.08^a^ | 0.29±  0.03^a^ | 0.28±  0.01^a^ | 0.23±  0.05^a^ | 0.22±  0.07^a^ | 0.22±  0.07^a^ |
| Intestinal body wall weight ratio (R, %) | 3.24±  0.96^a^ | 4.40±  1.09^a^ | 4.13±  0.44^a^ | 3.42±  1.12^a^ | 4.22±  0.37^a^ | 4.23±  0.55^a^ |
| Intestinal wall length ratio (RGL, %) | 3.65±  0.36^a^ | 3.95±  0.08^a^ | 3.71±  0.26^a^ | 3.42±  0.5^a^ | 4.22±  0.16^a^ | 4.23±  0.25^a^ |

Different letters show a significant difference (*P* < 0.05).
