# Supplementary material for: Effects of Undaria pinnatifida-derived brown algae polysaccharide (UPS) on the nutritional composition, digestive capacity, immune performance and intestinal microbiota of juvenile sea cucumber (Apostichopus japonicus)
Source: PeerJ. 2025 Aug 19;13:e19944. doi: 10.7717/peerj.19944 (PMC12372788; doi:10.7717/peerj.19944)
Supplement: Supplemental Information 2 [file peerj-13-19944-s002.docx]

| NO. | Sample | Top-hit taxon | Top-hit strain | Similarity (%) | Top-hit taxonomy | Completeness(%) |
| --- | --- | --- | --- | --- | --- | --- |
| 1 | HS0-1 | *Bacillus siamensis* | KCTC 13613 | 99.86 | Bacteria;Firmicutes;Bacilli;  Bacillales;Bacillaceae;  Bacillus | 95.7 |
| 2 | HS0-2 | *Bacillus siamensis* | KCTC 13613 | 99.79 | Bacteria;Firmicutes;Bacilli;  Bacillales;Bacillaceae;  Bacillus | 95.8 |
| 3 | HS0-5 | *Bacillus siamensis* | KCTC 13613 | 99.65 | Bacteria;Firmicutes;Bacilli;  Bacillales;Bacillaceae;  Bacillus | 95.9 |
| 4 | HS0-6 | *Staphylococcus epidermidis* | NCTC 11047 | 100 | Bacteria;Firmicutes;Bacilli;Bacillales;Staphylococcaceae;Staphylococcus | 95.2 |
| 5 | HS0-7 | *Bacillus siamensis* | KCTC 13613 | 99.86 | Bacteria;Firmicutes;Bacilli;  Bacillales;Bacillaceae;  Bacillus | 95.7 |
| 6 | HS1-3 | *Staphylococcus pasteuri* | ATCC 51129 | 99.86 | Bacteria;Firmicutes;Bacilli;Bacillales;Staphylococcaceae;Staphylococcus | 95.7 |
| 7 | HS1-5 | *Bacillus siamensis* | KCTC 13613 | 99.86 | Bacteria;Firmicutes;Bacilli;Bacillales;Bacillaceae;Bacillus | 95.8 |
| 8 | HS1-6 | *Bacillus*  *amyloliquefaciens* |  | 100 | Bacteria;Firmicutes;Bacilli;Bacillales;Bacillaceae;Bacillus | 96.4 |
| **9** | **HS1-7** | ***Bacillus***  ***subtilis*** |  | **100** | **Bacteria;Firmicutes;Bacilli;Bacillales;Bacillaceae;Bacillus** | **96.1** |
| 10 | HS1-8 | *Bacillus siamensis* | KCTC 13613 | 99.86 | Bacteria;Firmicutes;Bacilli;  Bacillales;Bacillaceae;  Bacillus | 96.1 |
| **11** | **HS2-2** | ***Bacillus***  ***subtilis*** |  | **100** | **Bacteria;Firmicutes;Bacilli;Bacillales;Bacillaceae;Bacillus** | **96.5** |
| 12 | HS2-6 | *Bacillus siamensis* | KCTC 13613 | 99.86 | Bacteria;Firmicutes;Bacilli;Bacillales;Bacillaceae;Bacillus | 95.7 |
| 13 | HS2-7 | *Staphylococcus epidermidis* | NCTC 11047 | 100 | Bacteria;Firmicutes;Bacilli;Bacillales;Staphylococcaceae;Staphylococcus | 95.7 |
| 14 | HS2-9 | *Bacillus siamensis* | KCTC 13613 | 99.86 | Bacteria;Firmicutes;Bacilli;Bacillales;Bacillaceae;Bacillus | 95.8 |
| 15 | HS2-10 | *Bacillus siamensis* | KCTC 13613 | 99.79 | Bacteria;Firmicutes;Bacilli;Bacillales;Bacillaceae;Bacillus | 95.9 |
| 16 | HS3-2 | *Bacillus velezensis* | CR-502 | 99.71 | Bacteria;Firmicutes;Bacilli;Bacillales;Bacillaceae;Bacillus | 96 |
| 17 | HS3-5 | *Bacillus siamensis* | KCTC 13613 | 99.86 | Bacteria;Firmicutes;Bacilli;Bacillales;Bacillaceae;Bacillus | 95.9 |
| 18 | HS3-6 | *Bacillus velezensis* | CR-502 | 99.85 | Bacteria;Firmicutes;Bacilli;Bacillales;Bacillaceae;Bacillus | 95.9 |
| 19 | HS4-1 | *Bacillus siamensis* | KCTC 13613 | 99.86 | Bacteria;Firmicutes;Bacilli;Bacillales;Bacillaceae;Bacillus | 95.7 |
| 20 | HS4-2 | *Bacillus siamensis* | KCTC 13613 | 99.86 | Bacteria;Firmicutes;Bacilli;Bacillales;Bacillaceae;Bacillus | 95.8 |
| 21 | HS4-3 | Staphylococcus pasteuri | ATCC 51129 | 99.86 | Bacteria;Firmicutes;Bacilli;Bacillales;Staphylococcaceae;Staphylococcus | 95.6 |
| 22 | HS4-6 | Bacillus siamensis | KCTC 13613 | 99.65 | Bacteria;Firmicutes;Bacilli;Bacillales;Bacillaceae;Bacillus | 96.1 |
| **23** | **HS4-8** | **Bacillus subtilis** |  | **99.72** | **Bacteria;Firmicutes;Bacilli;Bacillales;Bacillaceae;Bacillus** | **96.1** |
| 24 | HS4-11 | Bacillus siamensis | KCTC 13613 | 99.86 | Bacteria;Firmicutes;Bacilli;Bacillales;Bacillaceae;Bacillus | 96.2 |
| 25 | HS6-3 | Bacillus siamensis | KCTC 13613 | 99.86 | Bacteria;Firmicutes;Bacilli;Bacillales;Bacillaceae;Bacillus | 96.2 |
| 26 | HS7-1 | *Bacillus velezensis* | CR-502 | 99.71 | Bacteria;Firmicutes;Bacilli;Bacillales;Bacillaceae;Bacillus | 96 |
| 27 | HS5-2 | Bacillus velezensis | CR-502 | 99.85 | Bacteria;Firmicutes;Bacilli;Bacillales;Bacillaceae;Bacillus | 95.9 |
| 28 | HS5-5 | Bacillus siamensis | KCTC 13613 | 99.86 | Bacteria;Firmicutes;Bacilli;Bacillales;Bacillaceae;Bacillus | 95.7 |
