# Supplementary figures and images for: Effects of Undaria pinnatifida-derived brown algae polysaccharide (UPS) on the nutritional composition, digestive capacity, immune performance and intestinal microbiota of juvenile sea cucumber (Apostichopus japonicus)
Source: PeerJ. 2025 Aug 19;13:e19944. doi: 10.7717/peerj.19944 (PMC12372788; doi:10.7717/peerj.19944)

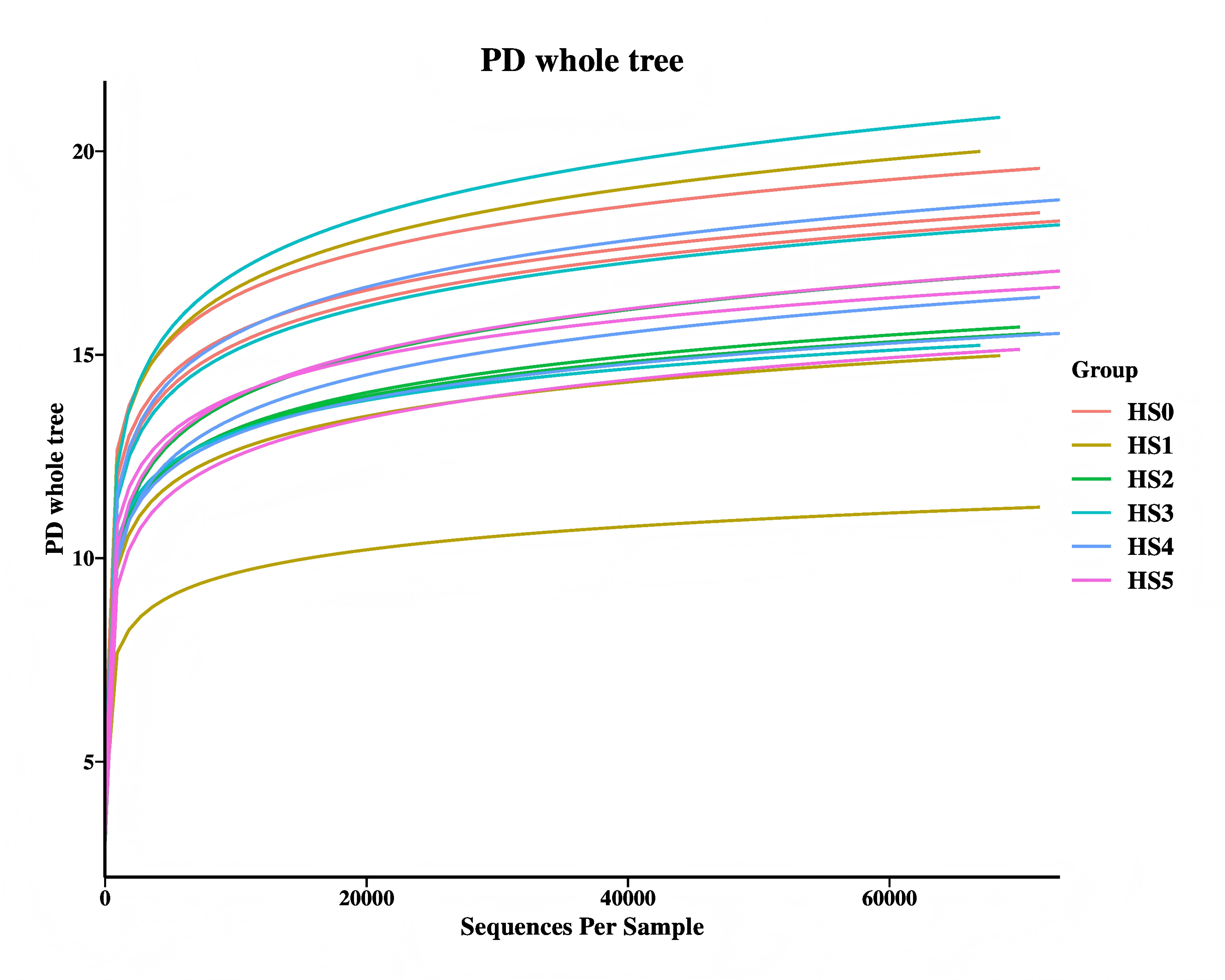

Supplement: Supplemental Information 3 [file peerj-13-19944-s003.png]

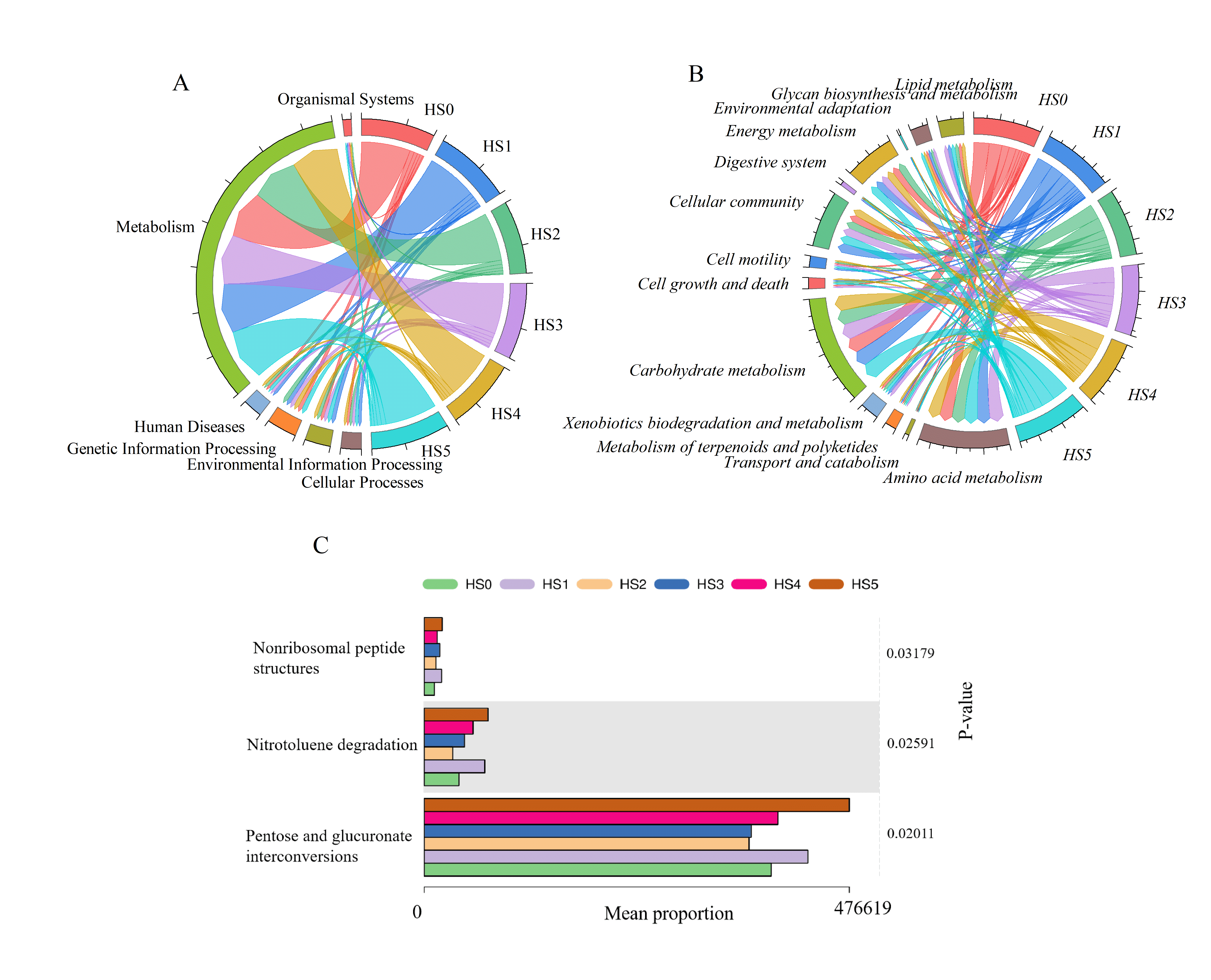

Supplement: Supplemental Information 4 [file peerj-13-19944-s004.png]
